# Effect of two corticotomy protocols on periodontal tissue and orthodontic movement

**DOI:** 10.1590/1678-7757-2019-0766

**Published:** 2020-07-03

**Authors:** Marcelo Lelis ZUPPARDO, Milton SANTAMARIA, Camila Lopes FERREIRA, Mariéllen LONGO, Joni Augusto CIRELLI, Mauro Pedrine SANTAMARIA, Maria Aparecida Neves JARDINI

**Affiliations:** 1 Universidade Estadual Paulista São José dos CamposSão Paulo Brasil Universidade Estadual Paulista (UNESP), Pós-graduação em Biopatologia Bucal, Curso de Odontologia - ICT, Disciplina de Periodontia, São José dos Campos, São Paulo, Brasil.; 2 UNIARARAS Centro Universitário Hermínio Ometto Programa de Pós-Graduação em Ortodontia Araras Brasil UNIARARAS, Centro Universitário Hermínio Ometto, Programa de Pós-Graduação em Ortodontia e Programa de Pós-Graduação em Ciências Biomédicas, Araras, Brasil.; 3 Universidade Estadual Paulista São José dos CamposSão Paulo Brasil Universidade Estadual Paulista (UNESP), Curso de Odontologia - ICT, Pos-Doutorado, Disciplina de Periodontia, São José dos Campos, São Paulo, Brasil.; 4 Universidade Estadual Paulista Faculdade de Odontologia AraraquaraSão Paulo Brasil Universidade Estadual Paulista (UNESP), Faculdade de Odontologia - FOAR, Disciplina de Periodontia, Araraquara, São Paulo, Brasil.; 5 Universidade Estadual Paulista São José dos CamposSão Paulo Brasil Universidade Estadual Paulista (UNESP), Curso de Odontologia - ICT, Disciplina de Periodontia, São José dos Campos, São Paulo, Brasil.

**Keywords:** Corticotomy, Orthodontic movement, Animal study

## Abstract

**Objective:**

To compare two corticotomy surgical protocols in rats to verify whether they alter conventional orthodontic movement.

**Methodology:**

Sixty Wistar rats were divided into three groups – orthodontic movement (CG), orthodontic movement and corticotomy (G1) and orthodontic movement with corticotomy and decortication (G2) – and euthanized after 7 and 14 days. Tooth movement (mm), bone volume fraction and bone volume ratio to total volume (BV/TV), and bone mineral density (BMD) were evaluated by micro-CT. The total amount of bone was measured in square millimeters and expressed as the percentage of bone area in the histomorphometry. The number of positive TRAP cells and RANK/RANKL/OPG interaction were also investigated.

**Results:**

Day 14 showed a statistically significant difference in orthodontic tooth movement in CG compared with G1 (7.52 mm; p=0.009) and G2 (7.36 mm; p=0.016). A micro-CT analysis revealed a difference between CG, G1 and G2 regarding BV/TV, with G1 and G2 presenting a lower BV/TV ratio at 14 days (0.77 and 0.73 respectively); we found no statistically significant differences regarding BMD. There was a difference in the total amount of bone in the CG group between 7 and 14 days. At 14 days, CG presented a significantly higher bone percentage than G1 and G2. Regarding TRAP, G2 had more positive cells at 7 and 14 days compared with CG and G1.

**Conclusion:**

Corticotomy accelerates orthodontic movement. Decortication does not improve corticotomy efficiency.

## Introduction

With an increasing number of adult patients seeking orthodontic treatment, orthodontists are constantly searching for treatments to accelerate orthodontic movement that are predictable and have few complications.^1^ Such techniques include reducing the treatment period using self-ligating bracket systems;^2^ wires with memory (NiTi);^3^ direct electric currents or magnet;^4^ micro implants;^5^ surgical interventions^6^ and administration of local or systemic medications.^7^

Among surgical interventions is corticotomy – an intentional bone injury limited to a cortical portion of the alveolar bone with minimal penetration into the medullary bone.^5^ It is indicated to speed up corrective orthodontic treatment and facilitate executing mechanically difficult orthodontic movement, as well as to correct moderate to severe skeletal occlusions and decrease treatment time.^6,8,9^

Fast orthodontic treatments are essential as shorter treatments are more acceptable to patients, and long-term treatments have been associated with negative results, such as an increased risk of dental cavities,^10^ periodontal disease,^11^ root resorption^12^ and pulp reactions.^13^

Accelerated osteogenesis has sparked interest in the orthodontic community and animal studies have demonstrated the biological reactions of bone remodeling and periodontal tissues when associated with corticotomy and orthodontic movement.^1^ During bone remodeling, a phenomenon known as regional acceleratory phenomenon (RAP) occurs. Frost^14^ (1983) described it as faster tooth movement due to reduced resistance of the cortical bone through the surgical procedure; Yaffe, Fine, Binderman^15^ (1994) described RAP as a temporary explosion of localized remodeling of soft and hard tissues, i.e. a regeneration that rebuilds the bone, thus restoring its normal state. Sebaoun, et al.^16^(2008) observed that RAP increases bone metabolism, activates osteoclasts and osteoblasts, and decreases bone density.

Baloul, et al.^17^ (2011) compared orthodontic movement with and without alveolar corticotomy, using tomography and molecular methods, and found that the corticotomy group achieved faster initial tooth displacement. Dibart, et al.^18^ (2014) used a minimally invasive technique (piezo-incision) that resulted in accelerated orthodontic movement and less extensive and traumatic surgical treatment.

The use of corticotomy in orthodontic treatment is increasing and studies have mainly investigated clinical cases with many variations in both surgical and orthodontic protocols.^9^ Corticotomy procedures can produce statistically and clinically meaningful temporary increases in the rate of orthodontic tooth movement with minimal side-effects.^19^

Thus, this study aimed to evaluate two surgical corticotomy protocols by an experimental model involving rats. The null hypothesis was that there is a similarity in orthodontic movement between two treatments with different amounts of surgical injury.

## Methodology

The study was approved by the CEUA nº 08/2015- ICT-SJC-UNESP. Sixty male Wistar rats (*Rattus norvegicus*, albinos) aged 90 days and weighing 300 g on average were kept in plastic cages, at room temperature (22ºC) for a 12-hour light cycle with standard diet and water *ad libitum.*

They were randomly divided into three groups: CG (n=20) received conventional orthodontic treatment; G1 (n=20) received a less invasive corticotomy treatment followed by orthodontic treatment; and G2 (n=20) received corticotomy with decortication and orthodontics, a more invasive protocol.

### Sample size

The sample size needed to determine tooth movement (primary outcome) calculated by a previous study^20^ with an alpha level of 5% and power of 80% was 17 animals/group. To ensure sufficient samples, the final n-sample comprised 20 animals/group.

### Orthodontic appliance placement

The orthodontic devices were installed on the animals’ lower first molar, as previously described.^21^ After anesthesia, a 0.12 mm alloy wire (Morelli, Sorocaba, SP, Brazil) was inserted between the first and second right molars. A closed-coil steel spring of 3 mm in length (Morelli, Sorocaba, SP, Brazil) was pre-calibrated so as to release a continuous force, and attached to the lower incisors with a light-curing resin (3M ESPE, USA), thus causing a spring pull of 40-g^22^ of force. A 40-g force was applied on the first molar (point of force application) and lower incisors (anchor point) to allow moving the first molar to the mesial direction.

### Surgical procedure

**Figure 1:**
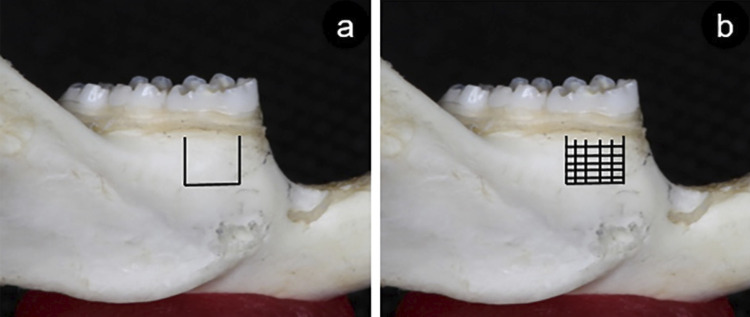
Schematic drawing of corticotomy(a) and decortication (b)

G1 and G2 animals underwent surgery just after installation of the orthodontic appliance. G1 received trichotomy of the masseter muscle region, and the area was disinfected with chlorhexidine digluconate. A 10-mm incision from the labial commissure region towards the mandible angle was performed, exposing the buccal cortical bone of the first lower molar roots.^23^ Corticotomy was performed using the tip (W1-0-CVDentus) of a piezo-surgical device (DentSurg CVDentus, Brazil) under saline irrigation, creating two vertical grooves and a horizontal one, with a U-shaped design in the apical region of the first lower molar (Figure1a).

G2 received corticotomy with a U-shape in the apical region of the first lower molar, with part of the cortical bone (decortication) being removed from the center of the groove (Figure 1b) with a spherical tip (R4). The muscle was repositioned and sutured with vicryl 4-0 and the skin was sutured with 3-0 silk.^21^

The animals were euthanized through transcardial perfusion at 7 and 14 days.^24^Their hemi-mandibles were removed and stored in a buffered 4% paraformaldehyde solution for 72 hours.

All analyses were evaluated by a blinded and previously calibrated examiner.

### Macroscopic analysis of orthodontic movement (mm)

We measured the amount of orthodontic movement at 7 and 14 days. Tooth movement from the mesial first molar to the distal third molar was quantified using a digital caliper (Mitutoyo Absolute Digimatic Model CD-6 “CX-B, Brazil) after dissecting the jaws.

### Computerized microtomography (micro-CT) analysis

CG, G1, and G2 hemi mandibles were scanned using a high-resolution microtomography (Sky Scan1176 Bruker MicroCT Actselaar Belgium). Sections of 9 µm thickness (50 ky, 500 µm) were made and reconstructed using the NRecon software (SKYcan, 2011,1.6.6.0). Misalignment compensation values were calculated individually.

The CTAnalyzer software delimited the region of interest using a grayscale (25–90 tons) which was used to evaluate changes in alveolar bone volume in the bifurcation root areas of the lower first molar in 3D slices. The parameters were determined according to Nogueira, et al.^25^ (2017): TV: total tissue volume measured by contours in the region of interest; BV: volume of mineralized tissue; BV/TV: bone volume fraction, ratio of bone volume to total volume; BMD: bone mineral density.

### Histomorphometric analysis

**Figure 2:**
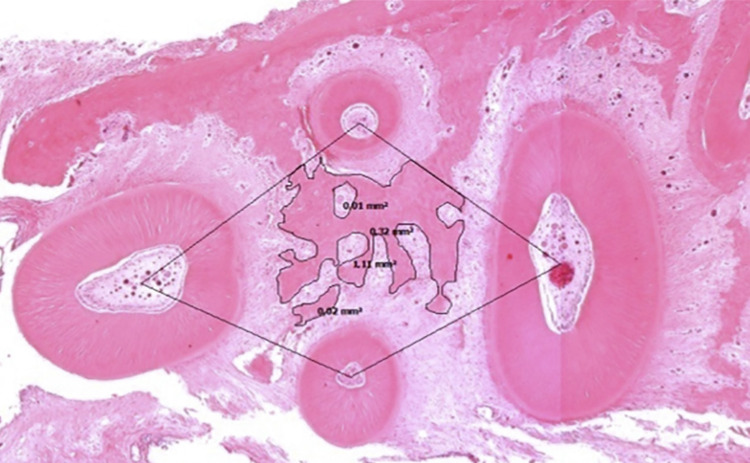
Delimitation of histomorphometric analyses Cross-section of the first molar mandibular with geometric area and total amount of bone outlined.

The samples were demineralized in a 10% EDTA solution at 7.8 pH and immersed in paraffin, then cut into transverse plane allowing visualization of the roots and the periodontal ligament of the first mandibular tooth. Five semi-sequential slices (5 µm) of the furcation region spaced 125 µm apart were stained with hematoxylin and eosin. Images were captured at 25x magnification using an Axioplan 2 microscope and Axiovision Rel 4.7 image capture software. Images were renamed to appropriately blind the examiner. Histomorphometric analysis was performed according to Dibart, et al.^18^(2014): a geometric area was delimited using the four center roots creating the interradicular area in which the bone tissue was measured in mm^2^ and the outcomes were expressed as percentages (Figure 2).

### Immunohistochemistry analyses (RANK, RANKL, OPG, and TRAP)

Before the immunohistochemical analyses, paraffin blocks were cut into serial cross-sections with 4 μm thickness and placed on slides previously treated with 3-aminopropyltriethoxysilane (Sigma Chemical, USA).

The samples (n=6/group/antibody) were immunohistochemically processed as previously described.^21^We performed indirect immunoperoxidase using the following primary antibodies: anti-receptor activator of nuclear factor-κB (anti-RANK, 1:100, ab13918, abcam, Cambridge, MA, USA), anti-receptor activator of nuclear factor kappa-B ligand (anti-RANKL,1:100, ab45039, abcam), anti-osteoprotegerin (anti-OPG,1:400; ab73400, abcam) and anti-tartrate resistant Acid Phosphatase (Anti-TRAP, 1:100; SC30833, Santa Cruz). For the negative control, one of the slices was incubated only with the antibody diluent (Spring-codADS-125). Positive control concerned the development of a golden-brown precipitate as the final product of the bone tissue reaction, specifically indicating osteoblasts.

A semi-quantitative immunolabeling analysis was performed for RANK, RANKL, and OPG antibodies. The region of interest was the mesial area of the vestibular root of the lower right first molar, i.e. the pressure region and adjacent to the corticotomy and zone of decortication. The established score criteria for these antibodies were applied according to previous studies^21^: score 0 – absence of immunolabeling; score 1 – low immunolabeling; score 2 – moderate immunolabeling; score 3 – high immunolabeling.

We defined TRAP immunolabeling as presence of brownish color in the cytosolic compartment of positive cells, which was performed by counting TRAP-positive cells located in the mesial area of the vestibular root (pressure region) and adjacent to the corticotomy and zone of decortication. The number of osteoclasts and pre-osteoclasts stained with TRAP was counted within the geometric center of the four roots of the first molar.^21^

All images were captured at 400x magnification using an Axioplan 2 microscope and Axiovision Rel 4.7 image capture software. The images were blinded and quantified using Image J.

### Statistics

Prior to each analysis, the examiner was calibrated using the Pearson test (k=0.89) and blinded to all groups. The statistical analyses were performed using Sigma Plot 12.0. Descriptive statistics consisted of mean and standard deviation, with sample normality verified by the Shapiro-Wilk test. The Mann-Whitney test was used for intragroup comparison concerning the macroscopic measurements of movement; the Kruskal-Wallis test was used for intergroup analysis. The micro-CT, histomorphometry and immunohistochemistry results were analyzed by two-way ANOVA at 5% significance with Tukey test for post-hoc analysis.

## Results

### Macroscopic measurement of orthodontic movement

Groups G1 and G2 showed statistically significant differences in orthodontic movement compared with CG, which received the orthodontic appliance only (p<0.01) (Table 1).

**Table 1:** Measurements of orthodontic movement in right mandibles measured in millimeters with a digital caliper


Period	CG	G1	G2
	RM	RM	RM

7 Days	6.98 ± 0.19^Aa^	7.12 ± 0.25^Aa^	7.18 ± 0.16^Aa^
14 Days	7.21 ± 0.13^Aa^	7.52 ± 0.36^Ab^	7.36 ± 0.13^Ab^

Uppercase letters: statistically significant difference in the intragroup comparison, Mann-Whitney. Lower case letters: statistically significant difference in the intergroup comparison, Kruskal-Wallis. RM: right mandible.

### Computerized microtomography

Regarding BV/TV, all groups showed no significant intragroup differences between 7 and 14 days; however, at 7 days, CG differed significantly from G2, and G1 was similar to CG and G2. At 14 days, CG differed significantly from G1 and G2. As for BMD, there were no significant inter- or intragroup differences (Table 2).

**Table 2:** Micro-CT measurements of bone mineral fraction and bone mineral density


		7 days		14 days	
	Groups	M±SD	Me	M±SD	Me

BV/TV	CG	0.73 ± 0.03^Aa^	0,72	0.87 ± 0.04^Aa^	0,88
	G1	0.77 ± 0.01^Aab^	0,77	0.77 ± 0.11A^Ab^	0,78
	G2	0.64 ± 0.11^Ab^	0,63	0.73 ± 0.03^Ab^	0,75
BMD	CG	0.82 ± 0.04^Aa^	0,81	0.95 ± 0.07^Aa^	0,97
	G1	0.90 ± 0.01^Aa^	0,9	0.92 ± 0.10^Aa^	0,94
	G2	0.80 ± 0.11^Aa^	0,81	0.87 ± 0.04^Aa^	0,88

Uppercase letters: statistically significant difference in intragroup comparison by two-way ANOVA. Lower case letters: statistically significant difference in intergroup comparison by two-way ANOVA. M: mean; SD: standard deviation; Me: median; BV/TV: bone volume/total volume; BMD: bone mineral density.

**Figure 3:**
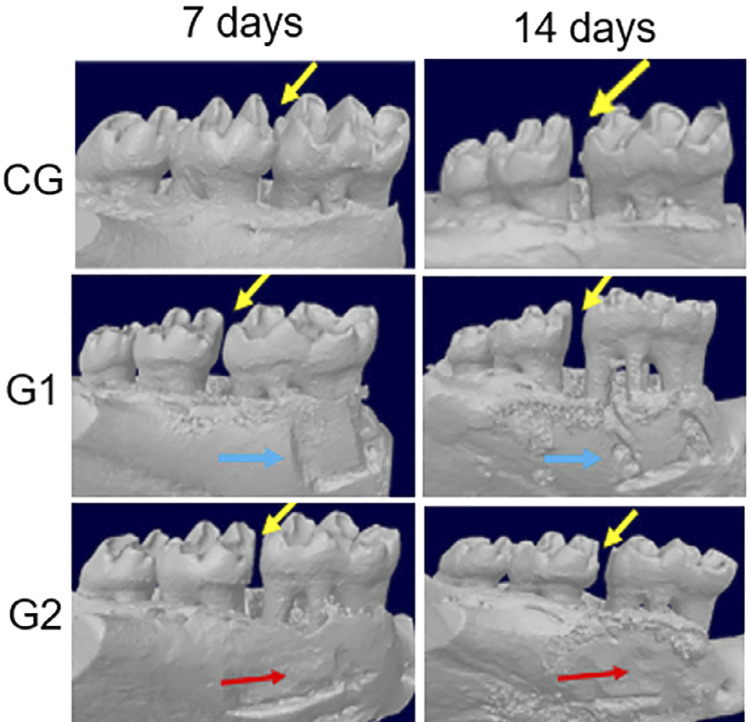
Transverse plane images performed on high-resolution microcomputer tomography Images represent the right mandible teeth, where corticotomy (G-blue arrow), and corticotomy and decortication (G2-red arrow) were performed. Yellow arrows indicate the space created between the first and second molars in the vestibular region of the transverse plane.

Figure 3 shows representative images of the first molar and region of interest obtained from the CG, G1, and G2 groups at 7 and 14 days.

### Histomorphometric analysis

The histomorphometric analysis revealed a significant difference in the total amount of bone present in CG at 14 days when compared with the 7^th^ day (p=0.001). Other groups exhibited no such behavior. The intergroup analysis showed no significant differences at 7 days; however, at 14 days, CG presented a significantly higher total amount of bone than G1 (p=0.003) and G2 (p=0.001). Regarding the percentage of bone volume, the intragroup analysis showed no differences between times in any of the groups. In the intergroup analysis, CG had a significantly higher percentage of bone volume than G1 (p=0.004) and G2 (p=0.002) at 14 days (Table 3).

**Table 3:** Histomorphometric measurements of the delineated geometric area in mm2


		7 days		14 days	
	Groups	M±SD	Me	M±SD	Me

Total amount of bone (mm^2^)	CG	0.35 ± 0.19^Aa^	0,38	0.54 ± 0.15^Ba^	0,6
	G1	0.35 ± 0.16^Aa^	0,35	0.41 ± 0.17^Ab^	0,38
	G2	0.36 ± 0.15^Aa^	0,38	0.40 ± 0.17^Ab^	0,43
% Bone	CG	38.3 % ± 17 %^Aa^	42%	49 % ± 11 %^Aa^	52%
	G1	33.9 % ± 13 %^Aa^	35%	39 % ± 12 %^Ab^	37%
	G2	35.2 % ± 13 %^Aa^	37%	38 % ± 15 %^Ab^	43%

uppercase letters: statistically significant difference in intragroup comparison by two-way ANOVA. Lowercase letters: statistically significant difference in intergroup comparison by two-way ANOVA. M: mean; SD: standard deviation; Me: median.

### Immunohistochemical analyses (RANK, RANKL, OPG, and TRAP)

**Figure 4:**
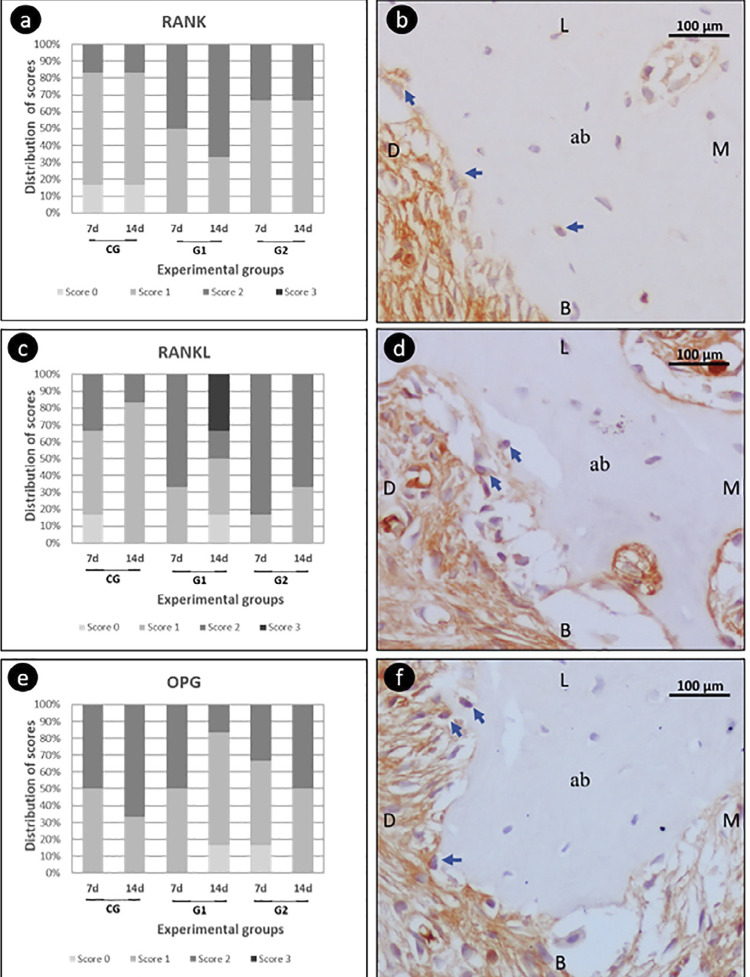
RANK, RANKL, OPG immunolabeling Immunolabeling score for RANK(a), RANKL(c), and OPG(e); photomicrographs evidencing the immunolabeling pattern for RANK(b); RANKL(d); and OPG(f). Blue arrows show immunolabeling cells. Abbreviations: ab: alveolar bone; L: lingual; B: buccal: M: mesial; D: distal 400x. Hematoxylin from Harris.

**Figure 5:**
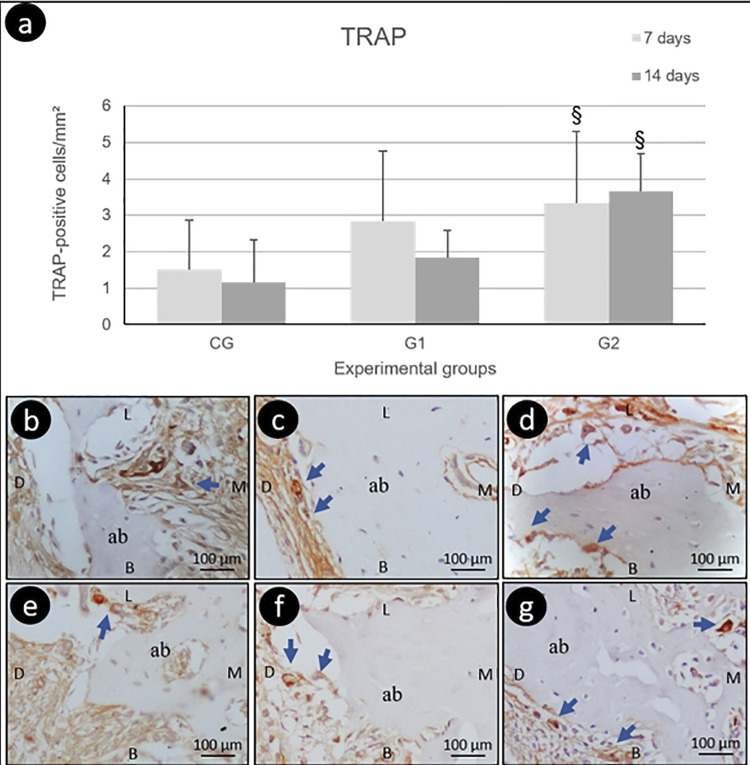
Mean and standard deviation of TRAP immunolabeling TRAP-positive cells/mm^2^ in different experimental groups and periods (a); photomicrographs evidencing the immunolabeling pattern for TRAP (blue arrows) in the CG 7d (b), CG 14d (c), G1 7d (d), G1 14d (e), G2 7d (f), G2 14d (g). Abbreviations: ab, alveolar bone; L: lingual; B: buccal: M: mesial; D: distal; §, statistically significant difference of CG and G1 groups in the same period. 400x. Hematoxylin from Harris.

In the immunohistochemical markers analysis, absolute values of RANK, RANKL, and OPG showed no significant differences in intra- and intergroup comparisons (Figure 4). In the TRAP analysis, G2 presented more positive cells, both at 7 days (p=0.04) and 14 days (p=0.04), compared with CG and G1 (Figure 5).

## Discussion

Some studies suggest that corticotomy be performed as a way of accelerating orthodontic movement to reduce total treatment period. Therefore, it was hypothesized that combining orthodontic movement with corticotomy surgery could increase the amount of tooth movement. We compared two surgical protocols: one with corticotomy and another with corticotomy and decortication, in an experimental model to evaluate the process of bone remodeling at the tomographic, histological and immunohistochemical levels.

We chose rats as an experimental model because this is a relatively well-established model due to its low cost, easy storage, good availability and easy logistics.^25^ However, some limitations must be carefully evaluated when extrapolating the results of an experimental model in rats to humans. The use of a stainless-steel spring resulted from the application of high physiological forces during the process of bone remodeling, since the loss of the initial part of the applied force allows a favorable reaction in the periodontal tissues, closer to that desired in clinical practice, and has been widely used in many experimental studies.^4,26,27,28^ An initial force of 40N is sufficient for tooth movement in this animal model and any loss of force intensity does not influence the analysis of tissue reactions in this experimental design.

The analysis of orthodontic movement in the different periods under study, whether associated with surgical procedures or not, revealed a statistically significant difference (p<0.01), confirming the validity of the experimental model in the literature concerning induced tooth movement.^27,28^ Assessing two time points during experimental tooth movement is important as the literature describes a drastic reduction of osteoclasts at 7 days and 14 days in the duration of a remodeling cycle.^29^ An electronic caliper helped to measure the linear displacement being an commonly used method,^21,27,30,31,32^ and micro-CT analyses evaluated bone microarchitecture and BMD.

In this study, both groups that received corticotomy experienced accelerated orthodontic movement, and although one surgical procedure was more invasive than the other, both showed a similar orthodontic movement rate. This may relate to the use of the piezo-incision, with its micrometric and selective slices; the piezo-electric blade allows safe and precise osteotomies with no osteonecrosis damage. Two systematic reviews^9,19^ showed that corticotomy can increase the orthodontic tooth movement rate due to an altered physiological response through the RAP with minimal side effects. Complications include root resorption, loss of tooth vitality, periodontal sequelae, dentin hypersensitivity, pain and swelling. More studies evaluating the side effects are needed to reach a solid consensus on its use.

The micro-CT analysis revealed no significant difference in the BV/TV between 7 and 14 days within any of the groups; but when we performed an intergroup comparison, both at 7 and 14 days, CG presented a higher BV/TV than G1 and G2. A decrease in BV/TV can be attributed to transient osteopenia, which occurs after bone lesions. Regarding BMD, there was no significant difference between groups, but the changes that occurred may suggest an increase in spinal cord space, an influx of inflammatory cells, and increased vascularization in the region.^17^

The similar BMD indicates that the observed bone pattern remained harmonic. Although BDM remained unaltered, the surgical procedure was sufficient to accelerate orthodontic movement in G1 and G2. These results corroborate a study conducted by Dibart, et al.^18^ (2014) who provided the first scientific evidence on the role of osteoclastic and osteoblastic activity associated with corticotomy and orthodontic movement.

The histomorphometric results were consistent with those shown by micro-CT images. We can understand the morphological processes by investigating biomarkers of bone remodeling and maturation.

Orthodontic forces promote cellular responses of hyalinization in the periodontal ligament, which induce bone resorption on the pressure side and bone deposition on the traction side.^28^ This process involves the induction of osteoclasts by the RANK-RANKL pathway and various inflammatory cytokines. RANKL/OPG binding favorably modulates osteoclastogenesis and is considered an important factor in controlling bone resorption.^33^

Based on the immunohistochemical analysis of RANK/RANKL/OPG biomarkers, our study showed greater expression of RANKL and its balance with OPG in animals that underwent corticotomy, despite the lack of biomarker differences, probably due to the time-length evaluated. Osteoblast activity was verified when the OPG was tested, and the maturation of osteoclasts with the analysis and quantification of TRAP in these cells.^34^ However, the animals showed greater maturation of osteoclasts and favored bone remodeling, as confirmed by the number of TRAP-positive cells, especially in G2.

This study suggests that although the most invasive technique (G2) resulted in the highest number of osteoclasts in the region of tooth movement, RANKL/OPG expression indicates a balance in the modulation of bone differentiation in both experimentally corticotomized groups. Allowing one to infer that the less invasive technique promoted similar clinical results regarding its biological effect and the clinical displacement of the tooth. Thus, the adverse effects of periodontal ligament hyalinization and subsequent presence of dental resorption can be minimized, even when performing more conservative corticotomy procedures, increasing tooth movement efficiency.

## Conclusion

According to our results, corticotomy accelerates orthodontic movement. Adding decortication to corticotomy does not improve its efficiency, suggesting that the less invasive technique should be selected for such purposes.
